# Working with communities to improve their eye health

**Published:** 2014

**Authors:** Islay Mactaggart

**Affiliations:** Research Fellow in Disability and Global Health, London School of Hygiene and Tropical Medicine, London, UK. Islay.Mactaggart@Ishtm.ac.uk

**Figure:**
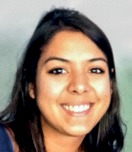
Islay Mactaggart

As an eye health worker you will be aware of various community interventions for improving eye health. These can involve **encouraging people to take better care of their eyes** (e.g. specific behaviour change programmes such as encouraging hand and face washing and improving infant feeding practices) and projects that **increase the number of people who make use of available services** (e.g. encouraging older people to come for cataract surgery, conducting outreach programmes, providing services in the community, or mass drug distribution programmes).

The key message of this issue is that improving eye health in the community can only be done in partnership with the community itself. There is no ‘one size fits all’! A service or intervention that has been successful in one community may very easily fail in another.

This is because each community is different, and there may be specific beliefs and traditions which will affect community members' willingness to change their habits or behaviour. There may also be particular barriers, unique to a particular community, that may limit the uptake of different services. It is therefore essential to first understand the community and then to involve them in the planning of interventions and services that suit their needs.

In some communities, a lack of knowledge, or risky behaviour that is considered socially acceptable, may increase people's risk of vision loss and blindness. This includes the behaviour of individuals (e.g. working in hazardous environments without eye protection) or the community as a whole (e.g. socially acceptable feeding practices that may lead to higher risk of vitamin A deficiency). It is important to fully understand these underlying issues before trying to make any changes and to work together with the community to challenge risky behaviour and promote good eye health behaviour.

**Figure:**
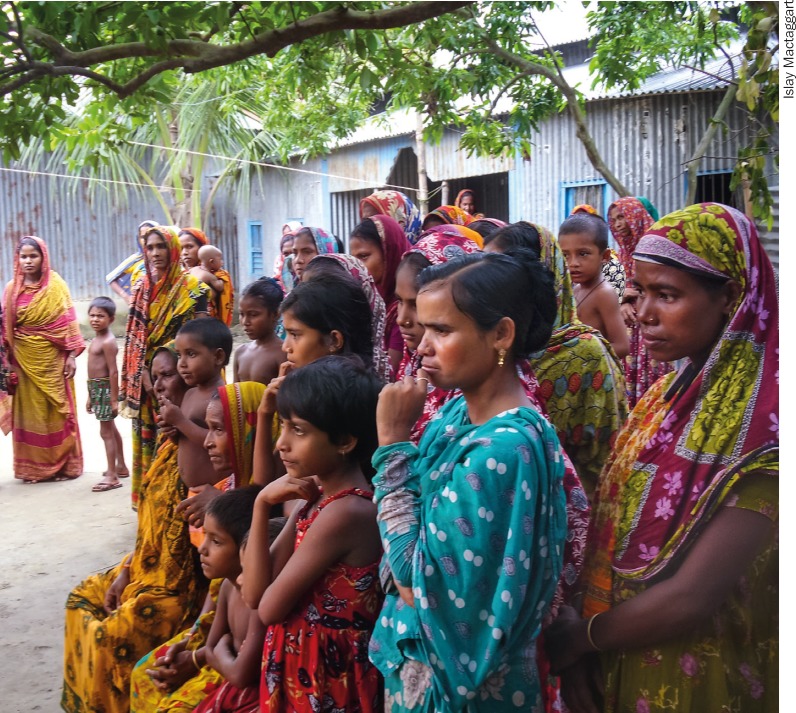
Community awareness raising session on available rehabilitation services. BANGLADESH

A second key way that you can improve eye health at the community level is to understand the potential barriers to uptake of services. There are many reasons why communities may not use eye health services, even when they are available. The barriers will vary from community to community (see the panel on page 63) and will require different solutions, depending on circumstances and what resources you have available.

Ways of overcoming these barriers, and improving the community's eye health, should be found in partnership with the local community.

Showing respect for the community and involving them in both the design and the implementation of services is crucial to any programme's success. This issue discusses how to involve all members of the community – including women and people with disabilities – in order to make sure that everyone's voice is heard. We will look at simple, effective ways in which you can empower the community to improve their eye health and work alongside them to deliver services that adequately meet their needs and that everyone is comfortable and confident using.

The first step will always be to understand the community, which is explored in detail in the article ‘How to empower communities to take action on improving eye health’ (page 64).

The article ‘Techniques to encourage people to take better care of their eye health’ (page 67) then gives specific examples of techniques you can use, depending on the type of services that you are hoping to provide.

**‘We look at simple, effective ways in which you can empower the community.’**

For example, if you are planning a mobile eye health clinic that will visit each village once a month then you might want to consider the ‘facilitating access’ section on how to make sure that there are no barriers to accessing the mobile clinic.

One potential barrier might be that people with mobility impairments may not be able to walk to the mobile clinic. Working together with the community, you might be able to come up with a solution, such as a list of people who need to be visited in their homes.

Another example might be that you wish to begin a mass drug distribution programme. Through discussion with the community, you may become aware of a general fear or mistrust in drugs, and it may be important to consider providing additional information on what the drug is and how it works to ensure uptake.

The issue also looks at ways in which you can measure the success, or impact, of the services that you provide (page 64) and make sure that they are meeting the needs of communities themselves.

This process does not involve a huge amount of work or resources, but helps you in terms of planning, in learning what works and what doesn't work, and in recording the actions that you have taken.

By the end of this issue, you will hopefully be better informed about the reasons why empowering communities and working with them to improve their eye health is so important, and about practical ways in which you can do this.

Including community approaches in your work has the potential to make sure that every community receives the best eye care possible, in a way that is tailored to their specific needs and situation.

Knowing what you, asa provider of eye care, should do, is of course important – as is providing high quality services. But to really improve eye health for everyone in the community, it is crucial to listen to communities, ask the right questions, understand their health-seeking behaviour and their knowledge about eye health, and work together with them on solutions.

**Barriers to the uptake of eye care services**

- **Poor marketing of eye care services,** including poor information about treatment costs. Patients will need to know about any related costs too, such as transport and accomodation.
- **Insufficient counselling of patients.** Incomplete information about causes and/or treatment options can lead to poor uptake of treatment. Information alone is not enough – patients need to be given time and support to help them make decisions (see page 69).
- **Traditional beliefs and stigma** about the causes of and treatments for particular eye conditions. These beliefs may contradict clinical explanations, and may even be dangerous if they lead to unhelpful or even harmful practices.
- **Decision making in the household.** If the household head makes the decisions on expenditure and time use for all members of the family, then women, the disabled or elderly household members may not be able to access the care they need.
- **Social barriers.** Stigma against particular ethnic minority groups, people with disabilities or people living with HIV/AIDS may discourage these groups from using available services for fear of abuse or non-acceptance by health care staff and other service users.
- **Not seeing the need.** Some people may not see the need for sight restoration or improvement.
- **Convenience and competing priorities.** Families with limited time or finances may feel that it is easier to visit a local (and potentially less costly) traditional healer easier than traveling for clinical services.
- **Inaccessibility.** Service centres may not be physically accessible for people with mobility impairments, and information may not be provided in an accessible way for people with hearing or visual impairments
- **Prior experiences.** Whether personal experience, or the experience of other members of their community, if someone has previously sought treatment from a service provider and had a bad experience, this can prevent others from coming forward.

**FROM THE FIELD**

**Working with the community in Cameroon**

**Okwen Marvice** is the resident ophthalmologist at Mbingo Baptist Hospital, a large NGO-funded hospital in the North-West of Cameroon. Patients come from across the country, and even from neighbouring countries Nigeria and Gabon, for treatment. The eye department conducts almost 15,000 eye consultations a year and provides in- and out-patient services, community outreach and school screening.

It is our duty to educate the community about common eye conditions. We attend village meetings and church services, collaborate with traditional rulers and local clinics to raise awareness, and give talks on community radio to educate the community about primary eye care and eye health. We focus on the following key messages, which are aimed at all community members:

- Have an eye check-up once a year, even if the eye is not painful (glaucoma is common in the area).
- Have regular check-ups if you have previously diagnosed with eye diseases; attend immediately if a problem with the eyes occurs.
- Avoid using traditional medicines or self-medication if you have an eye problem or a painful eye.
- Ensure children do not play with sticks or sharp objects.

We offer free eye check-ups at least once a year, train school teachers in visual acuity testing and in the identification and referral of children with eye problems. We also screen motorcycle riders for eye problems.

Our outreach services are organised with the help of field workers and volunteers and are sponsored by several international NGOs, including CBM and the International Response to Improve Sight (IRIS). Activities include performances during Glaucoma Week and World Sight Day, and community discussions in the local language, often led by former patients who tell the communities about their own experience attending the eye clinic. We believe that working in close collaboration with local health centres (who provide space for screening), community leaders, and social and religious groups, ensures that community members actively take part. This makes them more likely to change their behaviour.

Since the launch of the programme to reach out into the local communities and to educate them about eye care and where to access our services, the hospital has seen greater attendance for asymptomatic conditions, more people coming for annual screening, higher referral rates from teachers of children with eye conditions, and increased local knowledge about eye health.

*For further information about the work of Mbingo Baptist Hospital, visit*

**Figure:**
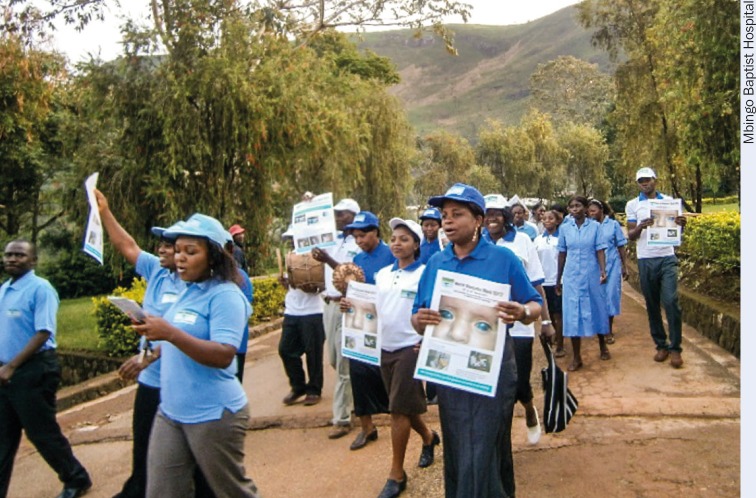
Volunteers spread awareness about glaucoma. CAMEROON

